# Prokaryotic Diversity in Aran-Bidgol Salt Lake, the Largest Hypersaline Playa in Iran

**DOI:** 10.1264/jsme2.ME11267

**Published:** 2011-12-15

**Authors:** Ali Makhdoumi-Kakhki, Mohammad Ali Amoozegar, Bahram Kazemi, Lejla Pašić, Antonio Ventosa

**Affiliations:** 1 Extremophile Laboratory, Department of Microbiology, School of Biology, College of Science, University of Tehran, 14155–6455 Qods, Tehran, Iran; 2 Cellular and Molecular Biology Research Center, Shaheed Beheshti University of Medical Sciences, Tehran, Iran; 3 Department of Biology, University of Ljubljana, Večna pot 111, 1000 Ljubljana, Slovenia; 4 Department of Microbiology and Parasitology, Faculty of Pharmacy, University of Sevilla, 41012 Sevilla, Spain

**Keywords:** *Archaea*, *Bacteria*, halophilic microorganisms, prokaryotic diversity, hypersaline lake

## Abstract

Prokaryotic diversity in Aran-Bidgol salt lake, a thalasohaline lake in Iran, was studied by fluorescence *in situ* hybridization (FISH), cultivation techniques, denaturing gradient gel electrophoresis (DGGE) of PCR-amplified fragments of 16S rRNA genes and 16S rRNA gene clone library analysis. Viable counts obtained (2.5–4 × 10^6^ cells mL^−1^) were similar to total cell abundance in the lake determined by DAPI direct count (3–4×10^7^ cells mL^−1^). The proportion of *Bacteria* to *Archaea* in the community detectable by FISH was unexpectedly high and ranged between 1:3 and 1:2. We analyzed 101 archaeal isolates and found that most belonged to the genera *Halorubrum* (55%) and *Haloarcula* (18%). Eleven bacterial isolates obtained in pure culture were affiliated with the genera *Salinibacter* (18.7%), *Salicola* (18.7%) and *Rhodovibrio* (35.3%). Analysis of inserts of 100 clones from the eight 16S rRNA clone libraries constructed revealed 37 OTUs. The majority (63%) of these sequences were not related to any previously identified taxa. Within this sampling effort we most frequently retrieved phylotypes related to *Halorhabdus* (16% of archaeal sequences obtained) and *Salinibacter* (36% of bacterial sequences obtained). Other prokaryotic groups that were abundant included representatives of *Haloquadratum*, the anaerobic genera *Halanaerobium* and *Halocella*, purple sulfur bacteria of the genus *Halorhodospira* and *Cyanobacteria*.

The hypersaline lake Aran-Bidgol covers an area of 2,400 km^2^ in the central part of Iran and is 1,000 km off the coast (Fig. 1). The lake is located at an altitude of 800 m in an area with an arid to semiarid continental climate. It was formed by the deposition of halite sediments from an ancient sea in different geological periods (Pliocene). In the wet season, these sediments are dissolved by rainfall (mean annual rainfall of 50–200 mm) and later subjected to high evaporation (mean annual evaporation of 1,727 mm) resulting both in elevated temperatures (up to 50°C, yearly fluctuations between 10°C–50°C) and high salinity of the brine. During the dry season, the salinity of the lake increases up to saturation, allowing for commercial production of halite.

The potential applications of halophiles in biotechnology (21, 29, 31) and advances in techniques for investigating microbial diversity have encouraged study of the microbial diversity of hypersaline environments in the past decade. Ecological studies were carried out in both athalasohaline and thalasohaline hypersaline environments, most often lakes (6, 8, 17, 22, 24) and saltern crystallizer ponds (5, 7, 20, 26, 27). In all studied environments, the microbial community was dominated by halophilic members of *Archaea* (often, but not always, *Haloquadratum walsbyi*), while their bacterial counterparts were scarce and often corresponded to members of *Bacteroidetes* (often *Salinibacter ruber*). Regardless of these similarities, the environments studied presented unique prokaryotic communities and were found to be a valuable source of novel prokaryotic diversity.

The following research focuses on microbial diversity in Aran-Bidgol salt lake and aims to a) describe its prokaryotic community using both cultivation and culture-independent approaches, b) compare the results obtained using both approaches, and c) discuss in more detail the specific characteristics of this hypersaline community.

## Materials and Methods

### Site description, samples collection and analysis

The Aran-Bidgol lake (34°18′–34°45′ N, 51°33′–52°10′ E, 2,400 km^2^) was sampled at the peak of the dry season (November 2007). We sampled shallow brine (up to 10 cm in depth) covering the playa at four different sites named according to brine color. These were B (black, 34°30′ N, 51°46′ E), G (green, 34°26′ N, 51°58′ E), O (orange, 34°39′ N, 51°53′ E) and R (red, 34°33′ N, 51°57′ E). The samples were collected in sterile plastic containers and kept in the dark at environmental temperature for four hours until analyzed in the laboratory. The salinity and pH of the samples were determined *in situ* with SevenMulti dual meter pH/conductivity (Mettler Toledo, Greifensee, Switzerland). Aliquots of the samples were sent to a commercial water chemistry laboratory (Khak-Azma, Iran) for analysis of chemical composition. Direct counts were obtained through DAPI staining. FISH experiments were performed as previously described (2, 34) using probes Arch915 (35) and EUB338 (1).

### Culture media and growth conditions

Halophiles were isolated under aerobic conditions on two growth media. The 23% MGM medium (7) had a total salt concentration of 23% (w/v) and contained (g L^−1^): NaCl 184.8, MgSO_4_·7H_2_O 26.9, MgCl_2_·6H_2_O 23.1, KCl 5.4 and CaCl_2_·2H_2_O 0.8, peptone 10.0, yeast extract 2.0, and agar 15.0; pH 7.2. Aran-Bidgol lake salt medium consisted of (g L^−1^): 230.0 Aran-Bidgol lake salt, peptone 10.0, yeast extract 2.0 and agar 15.0; pH 7.2. All samples were serially diluted up to 10^−6^ and plated according to Burns *et al.*(7). The plates were incubated aerobically at 40°C in sealed plastic containers for 8 weeks. An anisomycin (Santa Cruz Biotechnology, Santa Cruz, CA, USA) susceptibility test was carried out according to the disk diffusion method at a concentration of 30 μg per disk (28).

### DNA extraction and amplification of 16S rRNA genes

Haloarchaeal genomic DNA and environmental DNA were extracted as described previously (4, 7). Bacterial genomic DNA was extracted by the Genomic-DNA extraction kit (Roche, Diagnostic, Mannheim, Germany), according to the manufacturer’s recommendations.

Isolate 16S rRNA genes were amplified using either *Bacteria*-specific primer 5′-AGAGTTTGATCATGGCTCAG-3′ (19) or *Archaea*-specific primer 5′-TTCCGGTTGATCCTGCCGGA-3′ (10) in combination with the universal reverse primer 5′-GGTTACCT TGTTACGACTT-3′ (19). The PCR conditions were as follows. For *Archaea*: 94°C for 2 min, followed by 30 cycles of 94°C for 15 s, 51°C for 30 s and 72°C for 60 s, with final 7 min extension at 72°C; and for *Bacteria*: 94°C for 2 min, followed by 30 cycles of 94°C for 60 s, 55°C for 60 s and 72°C for 60 s, with final 7 min extension at 72°C. In amplifications involving environmental DNA, according to our experience, touchdown PCR was used where the annealing temperature ranged from 60°C–50°C, decreasing by 2 degrees every two cycles, followed by 20 cycles at 50°C.

For DGGE analysis, 16S rRNA genes were amplified using primers (25): 341F (5′-CGCCCGCCGCGCCCCGCGCCCGGCCCGCCGCCCCCGCCCC-CCTACGGGAGGCAGCAG) for *Bacteria* and 344F (5′-CGCCCGCCGCGCCCCGCGCCCGGCCCGCCGCCCCCGCCCC-ACGGGGCGCAGCAGGCGCGA) for *Archaea* and 907R (5′-CCGTCAATTCCTTTRAGTTT-3′) for both domains. The PCR program for both *Bacteria* and *Archaea* primer sets was: 94°C for 2 min, followed by 25 cycles of 94°C for 30 s, 55°C for 30 s and 72°C for 30 s, with final 5 min extension at 72°C.

### 16S rRNA gene library construction and Denaturing Gradient Gel Electrophoresis (DGGE)

PCR products of expected size (1,500 bp) were gel purified (DNA extraction kit; Roche, Germany) ligated into pGEM-T cloning vector (Promega, Madison, WI, USA) and used to transform *E. coli* DH5α cells. We constructed eight clone libraries. For each sampling site, a library of archaeal and bacterial 16S rRNA gene fragments was generated using pooled products of at least four independent PCRs. DGGE was performed with the DCode System (Bio-Rad, Hercules, CA, USA), as described previously by Mutlu *et al.*(24).

### Sequencing and sequence analysis

Sequencing was conducted on an ABI 3730XL DNA sequencer at Macrogen (Seoul, South Korea). Isolated 16S rRNA genes and DGGE bands were sequenced directly. Relevant sequences were extracted from GenBank (www.ncbi.nlm.nih.org) using BLASTN and through the EzTaxon server (9). Putative chimeric sequences were recognized using the Bellerophon server (15). The sequences were considered to belong to an operational taxonomic unit (OTU) if they shared ≥97% sequence identity.

The alignments were generated using MUSCLE web server (http://www.ebi.ac.uk/Tools/msa/muscle/). Maximum likelihood searches under a general-time-reversible (GTR) substitution model with gamma distributed rate heterogeneity and a proportion of invariable sites (GTR + Γ + I ) were performed using MEGA ver. 5 (36). Topology support was assessed using non-parametric bootstrapping. Rarefaction and ∫-LIBSHUFF analyses were performed using computer software MOTHUR (32).

### Accession numbers

The sequences were deposited in the GenBank sequence database under accession nos. HQ425031–HQ425248.

## Results

### Sample characteristic, total DAPI cell count and FISH analysis

The physicochemical properties of water samples collected and cell counts are presented in Table 1. We identified Na^+^ and Cl^−^ major ions in the samples, which were followed in abundance by SO_4_^2−^ and Mg^2+^. DAPI cell counts were comparable in all samples studied and ranged 3.4–4.1 × 10^7^ cells mL^−1^. Cells hybridizing with *Archaea*-specific probes and *Bacteria*-specific probes represented 50%–75% and 18%–37% of detected cells, respectively.

### Diversity of microorganisms isolated from Aran-Bidgol lake

After eight weeks of incubation, viable counts obtained on two media used were comparable and ranged 2.5–4 × 10^6^ CFU mL^−1^. We isolated 813 isolates and analyzed a random subset of 112 isolates; 101 *Archaea* and 11 *Bacteria* as determined based on their anisomycin susceptibility. All strains were cultured on 23% MGM media.

**Fig. 2:**
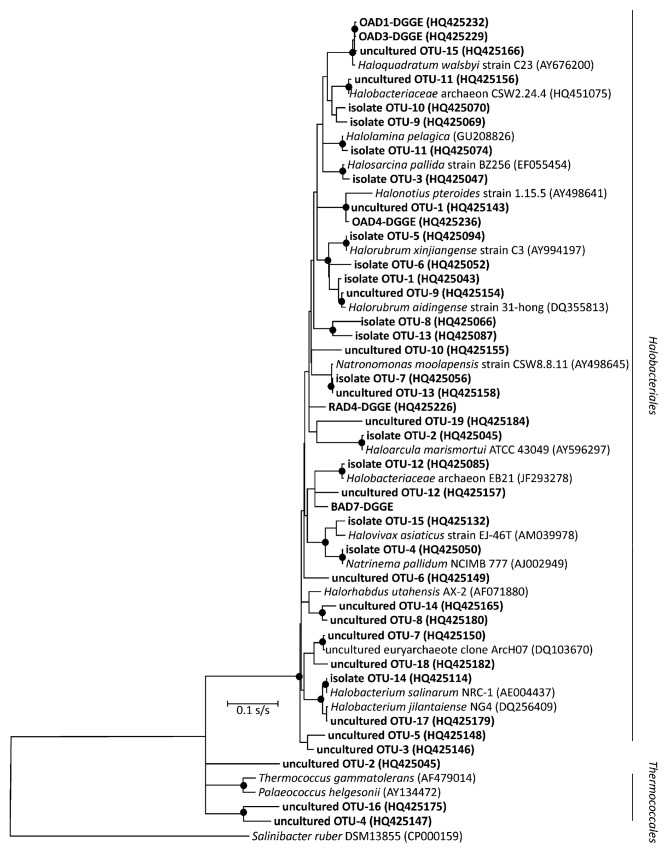
Phylogenetic reconstruction of 16S rRNA of archaeal sequences recovered from Aran-Bidgol lake. The most likely topology shown here was obtained under the General-Time-Reversible substitution model with gamma distributed rate heterogeneity and a proportion of invariable sites (GTR + Γ + *I*). Scale represents the expected number of substitutions per site. Significantly supported nodes are marked with bullets.

**Fig. 3:**
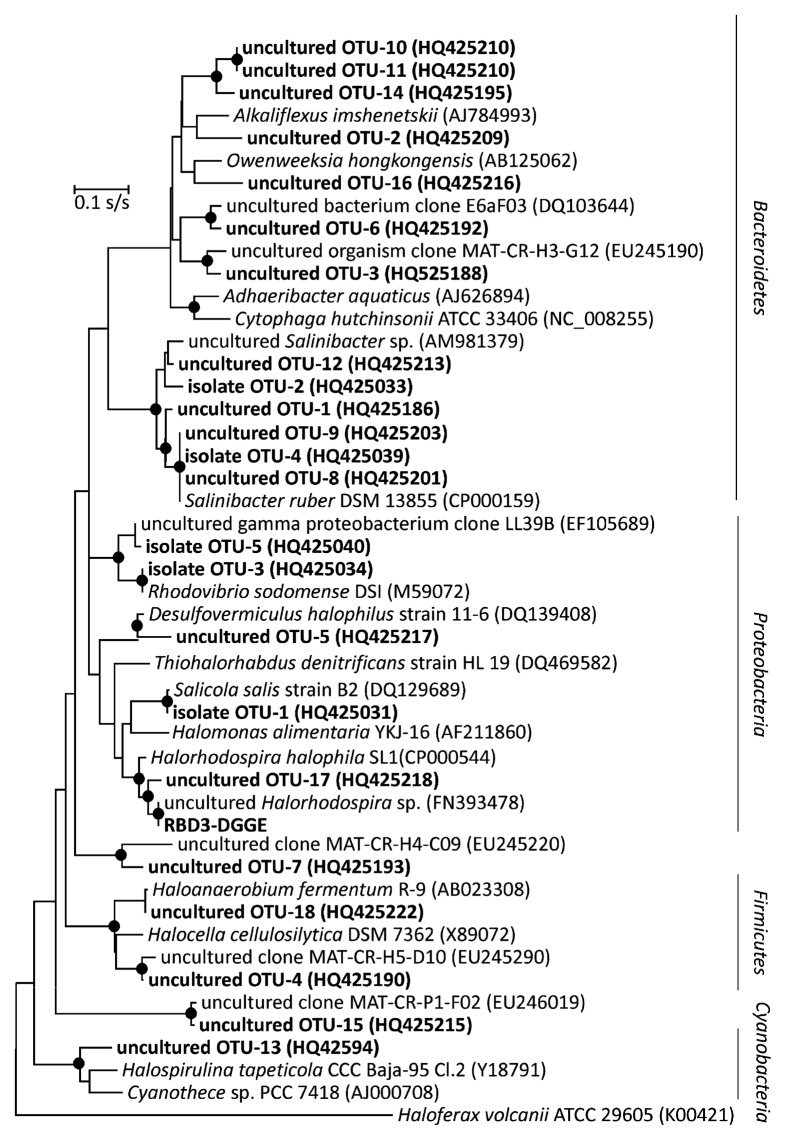
Phylogenetic reconstruction of 16S rRNA of bacterial sequences recovered from Aran-Bidgol lake. The most likely topology shown here was obtained under the General-Time-Reversible substitution model with gamma distributed rate heterogeneity and a proportion of invariable sites (GTR + Γ + *I*). Scale represents the expected number of substitutions per site. Significantly supported nodes are marked with bullets.

Archaeal isolates belonged to *Halobacteriacae* and formed 15 OTUs (Fig. 2, Table 2). These were phylogenetically related to the genera *Halorubrum* (55.4% of isolates obtained), *Haloarcula* (17.8%), *Natrinema* (4.0%), *Halogeometricum* (3.0%), *Natronomonas* (3.0%), *Halobacterium* (2.0%), *Halovivax* (2.0%), *Halolamina* (2.0%) and *Halorientalis* (1%). The remaining 10% of haloarchaeal isolates were phylogenetically unrelated to any previously cultivated taxa and are candidates for new genus-level and species-level taxa in the family *Halobacteriaceae* (Fig. 2). Bacterial isolates clustered into 5 OTUs (Fig. 3, Table 2), and were phylogenetically related to the following genera: *Rhodovibrio* (35.3% of bacterial isolates obtained), *Salinibacter* (18.7%) and *Salicola* (18.7%). The remaining OTUs (27.3%) were phylogenetically unrelated to any previously cultivated bacterial taxa and shared ≤93% sequence identity with known cultivated species.

### Sequence analysis of environmental 16S rRNA genes recovered from Aran-Bidgol lake

We randomly selected and sequenced a sample of 50 bacterial and 50 archaeal clones from eight 16S rRNA libraries constructed. We removed eleven chimeric sequences, assigned sequences to OTUs and performed phylogenetic analysis (Figs. 2 and 3). Environmental sequences of *Archaea*, which formed 19 OTUs, yielded substantial novelty. We identified four groups also detected by the cultivation approach and related to the genera *Halorubrum* (5% of sequences recovered), *Haloarcula* (5%), *Natronomonas* (5%) and *Halobacterium* (5%); however, 16% of the sequences branched with the member of *Halorhabdus* with whom they shared 93% sequence similarity. This group was followed in abundance by phylotypes related to *Haloquadratum* (10% of sequences analyzed). The 48% of the recovered sequences belonging to *Halobacteriaceae* was unrelated to any previously reported sequences. In addition, 18% of the obtained sequences branched independently within *Euryarchaea*. These sequences were very different from other sequences in the databases and were most similar to members of *Thermococcales* with whom their shared 81.3%–82.6% sequence similarity.

**Fig. 4:**
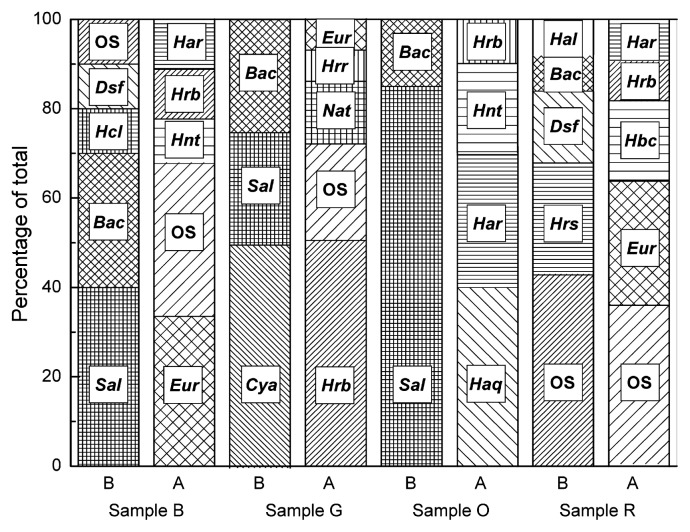
Phylotype diversity for *Archaea* and *Bacteria* in Aran-Bidgol lake. The bar chart compares 16S rRNA sequence diversities of phylotypes recovered from four sampling sites (sites B, G, O, and R) at the lake. Abbreviations: *Bacteria: Sal: Salinibacter; Bac*: other *Bacteroidetes; Hcl: Halocella; Dsf: Desulfovermiculus; Cyn: Cyanobacteria; Hrs: Halorhodospira*, *Hal: Halanaerobium. Archaea: Eur: Euryarchaea* other than *Halobacteriales; Hnt: Halonotius*, *Hrb: Halorhabdus; Har: Haloarcula; Nat: Natronomonas*, *Hrr: Halorubrum; Haq: Haloquadratum; Hbc: Halobacterium*, *OS*: Other sequences not affiliated with identified taxa.

Within the bacterial clone library we recovered members of three bacterial phyla. As observed in the cultivation approach, environmental sequences related to *Bacteroidetes* dominated the samples and represented 59% of total sequences recovered. Most of these sequences belonged to the genus *Salinibacter* (40%), but also appeared to form a novel lineage within this group. *Proteobacteria* representatives included *Gammaproteobacteria* such as purple sulfur bacteria of the genus *Halorhodospira* (7% of sequences recovered) and also halophilic sulfur reducing *Deltaproteobacteria* of the genus *Desulfovermiculus* (7%). *Firmicutes* represented 7% of sequences recovered and were affiliated with the anaerobic genera *Halanaerobium* and *Halocella*. Interestingly, 9% of the sequences recovered were affiliated with *Cyanobacteria*. Finally, 11% of sequences were not affiliated with any identified taxa. ∫-LIBSHUFF analysis of clone library sequences suggested that there is a high probability (*P* < 0.001) that the libraries constructed at different sites contain different taxonomic lineages. The assemblages found at each sampling site are presented in Fig. 4. Black brine (sample B) had the lowest pH among sampling sites (pH 6.7) and the highest sulfate and Fe^2+^ ion concentrations (Table 1). Clones related to heterotrophic sulfate-reducing bacteria were recovered from libraries constructed from this site. Almost half of the clones in the bacterial library constructed from green sample G were related to *Cyanobacteria*, which are probably responsible for the observed color, although the likely presence of eukaryotic autotrophs (e.g. *Dunaliella*) should not be neglected. In both samples, *Bacteria* were unusually abundant and represented 32% and 37% of total cell counts, respectively. Orange pigmented sample O had comparatively highest concentrations of Mg^2+^ and K^+^. *Archaea* strongly dominated this sample (74% of total cell counts), while bacterial clones were affiliated almost exclusively with *Salinibacter*. Finally, the red sample supported phylotypes related to red pigmented autotrophic bacteria, heterotrophic anaerobic bacteria and halophilic archaea.

**Fig. 5:**
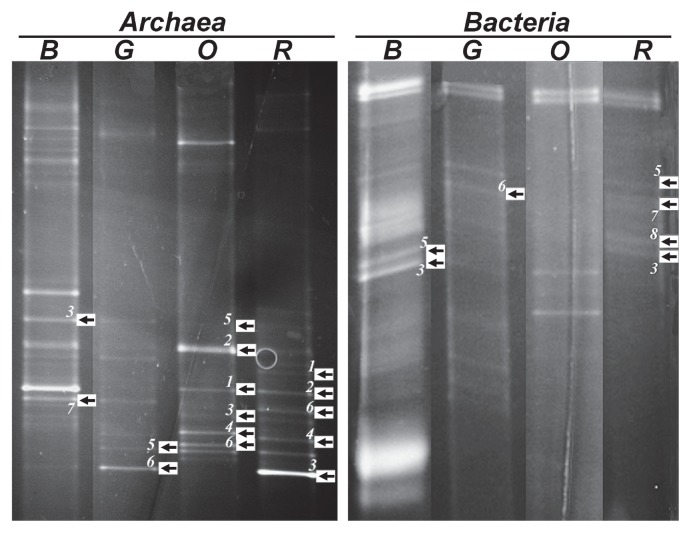
DGGE analysis of archaeal (left) and bacterial (right) diversity in Aran-Bidgol. Samples from four sampling sites (B, G, O, and R) indicated in separate column.

Regarding DGGE analysis, selected bands were obtained from the gels, reamplified and sequenced. Fig. 5 shows the DGGE patterns and bands that could be successfully sequenced, labeled with arrows. Overall, 40% of the archaeal sequences were related to *Haloquadratum walsbyi* following the sequences related to *Halorubrum* (25%), *Halonotius* (12.5%), *Halorhabdus* (12.5%) and *Haladaptatus* (10%). All analyzed bacterial bands were affiliated to *Halorhodospira* (Table 3).

**Fig. 6:**
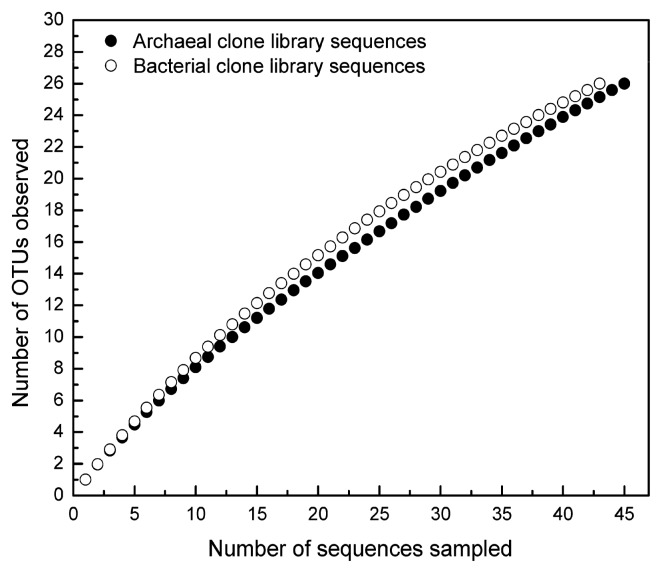
Rarefaction curve of obtained archaeal (filled circle) and bacterial (empty circle) sequences determined at a distance of 3% as implemented in MOTHUR (32) using 16S rRNA gene clone library sequences from Aran-Bidgol lake.

We applied rarefaction to evaluate whether the screening of 100 16S rRNA gene clones obtained from Aran-Bidgol lake was sufficient to estimate prokaryote diversity within our clone libraries (Fig. 6). For both domains, rarefaction curves did not reach a clear plateau, suggesting that additional sequencing would have revealed further diversity.

## Discussion

Iran has a great diversity of hypersaline environments whose microbial population needs to be elucidated. Aran-Bidgol salt lake is the largest hypersaline seasonal playa in Iran. According to its physicochemical properties, this inland lake was classified as thalassohaline. We sampled this lake during the dry season in areas which remain covered with up to 10 cm of brine. The brine ionic composition reflected that of seawater: Na^+^ was the dominant cation, Cl^−^ was the dominant anion, followed by SO_4_^2−^ and the pH was about neutral. In addition, the lake was found to have a high concentration of Mg^2+^ which surpasses the concentrations measured in some thalasohaline hypersaline lakes studied by order of magnitude (22, 24). Total cell counts in the lake (3–4 × 10^7^ cells mL^−1^) were in the range of the microbial populations found in other similar environments studied (10^6^–10^7^ cells mL^−1^) (13, 22, 24).

In contrast to saline alkaline lakes, where cultivated diversity is dominated by members of *Bacteria*(14, 23), the lake brine was found to be dominated by archaeal cells; however, compared to other neutral hypersaline lakes studied (3, 22, 30), the proportion of *Bacteria* in the lake was unusually high. In fact, bacterial counts in a similar range have only been reported from hypersaline lake Tuz in Turkey (24).

Many isolates were related to the genera *Halorubrum*, *Haloarcula* and *Haloferax*, as could be expected, given the ease with which they thrive under laboratory conditions rather than of their high abundance in the habitat (13). We tried to isolate *Haloquadratum*-related species by using an extinction-dilution method but were only able to obtain enrichment cultures of this species. Ten archaeal isolates were not affiliated to any identified taxa (≤92% similarity).

Isolates belonging to *Bacteria* represented 10% of the analyzed strains. We identified moderate halophiles of the genus *Rhodovibrio* and extremely halophilic *Salicola salis*, a non-pigmented bacterium originally isolated from a sabkha in Algeria (18) as well as strains of *Salinibacter ruber*. In addition, we obtained another group of isolates most closely related to *Salinibacter ruber*, but sharing only 93.5% 16S rRNA gene sequence similarity. We assume that these isolates could represent either another species within the genus *Salinibacter* or perhaps a novel genus within ‘*Rhodothermaceae*’. We further hypothesize that additional bacterial diversity could be obtained by altering the growth conditions used. These suppressed the growth of anaerobic, autotrophic and some probably moderate halophilic members of *Bacteria*, which were found abundant in clone libraries.

The main components of the Aran-Bidgol lake microbial community as depicted by PCR-based approaches were *Bacteroidetes* and *Halobacteriales*. All archaeal clones recovered belonged to *Euryarchaeota*, most frequently to *Halorhabdus* spp., originally isolated from Great Salt Lake in Utah (37), although several other groups were abundant, including a group of clones which formed a deep branch within the *Euryarchaeata* and did not cluster with any previously identified sequences. Similar assemblages have not been previously reported. Indeed, the archaeal community composition differed in hypersaline lakes studied and was often composed of novel and deeply branching euryarchaeal sequences. The majority of bacterial phylotypes was related to *Bacteroidetes*, most often to *Salinibacter ruber*, a phenomenon previously observed in neutral hypersaline lakes (22, 24). We also recovered members of *Gammaproteobacteria*, found to constitute an important component in both saline and alkaline lakes (12, 17, 22). We were very surprised to recover phylotypes related to *Cyanobacteria* (8% of clones recovered) at studied salinities (30–33%). Thus, it would be interesting to determine whether these organisms are also metabolically active. Phylotypes related to *Firmicutes* represented 6% of all OTUs observed in our study. This is somewhat surprising as the fluctuations between lake and dry playa environments should select for endospore-forming taxa. Indeed, these phylotypes represented 11–25% of all OTUs observed in the majority of saline and alkaline lakes studied (17, 23, 33). Similar proportions of *Firmicutes* were reported from athalassohaline and neutral Lake Chaka (7% of clones recovered), while no *Firmicutes*-related phylotypes were reported from Atacama desert lakes (11).

The comparison among the three methods for the study of microbial diversity in this lake revealed that culture-independent methods showed higher diversity than cultivation, by harboring 70% and 60% unique sequences for *Archaea* and *Bacteria*, respectively. DGGE seems not to be an effective method for the study of the microbial population in this lake, as presented by only 6% unique sequences for *Archaea* and no unique sequences for *Bacteria*. Only one group, *Halorubrum*, was detected by all three methods. The combination of a polyphasic approach consisting of cultivation- and culture-independent methods gives a good description of the prokaryotic diversity in hypersaline environments.

In conclusion, half of the sequences obtained in this study were related to groups previously obtained both from neutral and alkaline saline lakes (11, 16, 17, 23, 24). Thus, in spite of local-specific organisms, both saline and alkaline lakes appear to support microbial communities similar in composition, but differing in community structure.

## Figures and Tables

**Fig. 1:**
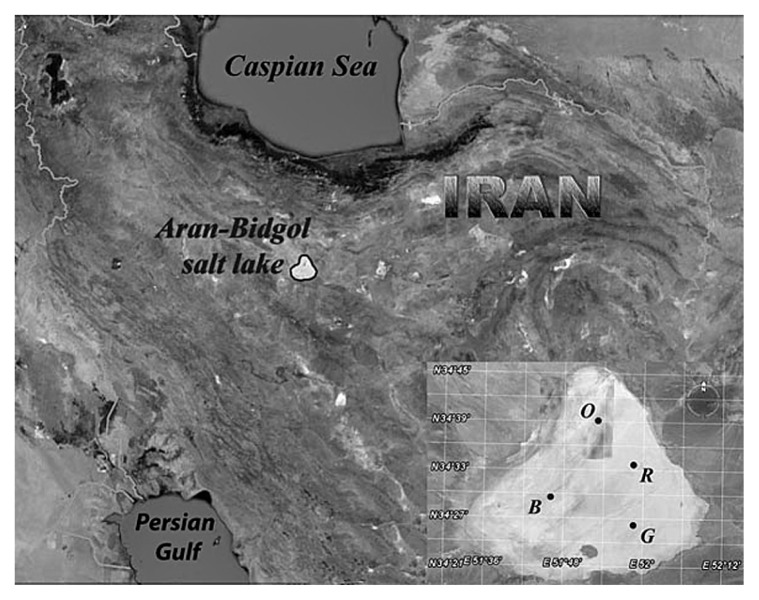
Location of the Aran-Bidgol lake and sampling sites (B, G, O, and R) used in this study.

**Table 1:** Physico-chemical properties of water samples from Aran-Bidgol lake, total DAPI cell count and relative percentages of hybridized cells with specific probes

Site	S^a^ (%)	pH	T (°C)	Ion concentration (g L^−1^)									DAPI count^b^ (10^7^ cells mL^−1^)	% of total FISH counts for probes	
	
				Na^+^	Mg^2+^	Ca^2+^	K^+^	Mn^+^	Fe^2+^	Cl^−^	SO_4_^2−^	HCO_3_^−^		ARC915	EUB338
B	31	6.7	38	88.4	7.8	0.27	2.6	0.0006	0.0005	158.3	4.2	0.002	3.9 ± 0.4	63	32
G	32	7.0	38	84.8	9.2	0.27	2.7	0.0010	0.0001	157.6	4.1	0.003	4.1 ± 0.4	55	37
O	30	7.3	38	78.3	12.1	0.20	3.7	0.0002	<0.0001	157.0	3.8	0.004	3.4 ± 0.5	74	18
R	33	6.9	38	88.4	8.8	0.28	2.5	0.0004	0.0002	162.2	4.1	0.005	4.1 ± 0.4	71	25

a Salinity as measured by hand refractometer

b Numbers refer to mean number of cells mL^−1^ ± standard deviation

**Table 2:** Comparison of isolate 16S rRNA sequences obtained from four sampling sites on 23% MGM media with those available in EzTaxon (9)

OTU-97%	No. of isolates	Closest identified species	Similarity (%)
*Archaea*			
1	34	*Halorubrum kocurii* BG-1 (AM900832)	98.5
2	18	*Haloarcula vallismortis* CGMCC1.2048 (BEF645688)	98.9
3	3	*Halogeometricum borinquense* DSM 11551 (ABTX01000001)	98.9
4	4	*Natrinema pallidum* NCIMB 777 (AJ002949)	98.8
5	22	*Halorubrum chaoviator* Halo-G (AM048786)	99.7
6	3	*Halorubrum luteum* CGSA15 (DQ987877)	93.6
7	3	*Natronomonas moolapensis* 8.8.11 (AY498645)	97.8
8	1	*Halorientalis regularis* JCM 16425 (GQ282621)	98.6
9	1	*Halogeometricum borinquense* DSM 11551 (ABTX01000001)	93.7
10	3	*Halosarcina pallida* BZ256 (EF055454)	93.5
11	2	*Halolamina pelagica* TBN21 (GU208826)	98.3
12	2	*Halobiforma lacisalsi* AJ5 (AY277582)	91.8
13	1	*Halalkalicoccus tibetensis* DS12 (AF435112)	89.5
14	2	*Halobacterium salinarum* NRC-1 (AE004437)	99.9
15	2	*Halovivax asiaticus* EJ-46 (AM039978)	96.4
*Bacteria*			
1	2	*Salicola salis* B2 (DQ129689)	99.4
2	2	*Salinibacter ruber* DSM 13855 (CP000159)	93.0
3	4	*Rhodovibrio sodomensis* DSI (M59072)	98.9
4	2	*Salinibacter ruber* DSM 13855 (CP000159)	99.5
5	1	*Rhodovibrio sodomensis* DSI (M59072)	92.8

**Table 3:** Comparison of DGGE bands sequences with those available in EzTaxon (9)

DGGE band	Closest identified taxon	Similarity (%)
*Archaea*		
RAD4	*Halorubrum litoreum* Fa-1 (EF028067)	92.7
RAD6	*Halorubrum kocurii* BG-1 (AM900832)	91.2
RAD2	*Haloquadratum walsbyi* C23 (AY676200)	99.4
OAD3	*Halonotius pteroides* CSW1.15 (AY498641)	95.2
OAD5	*Haloquadratum walsbyi* C23 (AY676200)	98.8
RAD1	*Haloquadratum walsbyi* C23 (AY676200)	99.0
OAD1	*Haloquadratum walsbyi* C23 (AY676200)	98.6
BAD3	*Haloquadratum walsbyi* C23 (AY676200)	98.6
RAD3	*Halorubrum alkaliphilum* DZ-1 (AY510708)	97.8
OAD2	*Haloquadratum walsbyi* C23 (AY676200)	97.8
OAD4	*Halonotius pteroides* CSW1.15 (AY498641)	95.0
OAD6	*Halorubrum arcis* AJ201 (DQ355793)	97.6
BAD7	*Haladaptatus cibarius* D43 (EF660747)	93.2
GAD5	*Halorhabdus tiamatea* SARL4B (EF127229)	95.7
GAD6	*Halorhabdus tiamatea* SARL4B (EF127229)	95.8
*Bacteria*		
RBD7	*Halorhodospira halophila* DSM 244 (M26630)	95.0
RBD8	*Halorhodospira halophila* DSM 244 (M26630)	95.1
RBD5	*Halorhodospira halophila* DSM 244 (M26630)	95.5
GBD6	*Halorhodospira halophila* DSM 244 (M26630)	95.0
BBD3	*Halorhodospira halophila* DSM 244 (M26630)	89.8
BBD5	*Halorhodospira neutriphila* SG 3301 (AJ318525)	90.0
RBD3	*Halorhodospira halophila* DSM 244 (M26630)	95.0
